# Time-Location Patterns of a Population Living in an Air Pollution Hotspot

**DOI:** 10.1155/2010/625461

**Published:** 2010-04-22

**Authors:** Xiangmei (May) Wu, Zhihua (Tina) Fan, Pamela Ohman-Strickland

**Affiliations:** ^1^Environmental and Occupational Health Sciences Institute (EOHSI), University of Medicine and Dentistry of New Jersey (UMDNJ) and Rutgers University, Piscataway, NJ 08854, USA; ^2^Department of Public Health, University of California, Davis, CA 95616, USA; ^3^Environmental and Occupational Medicine, Robert Wood Johnson Medical School, UMDNJ, NJ 08854, USA; ^4^Department of Biostatistics, School of Public Health, UMDNJ, NJ 08854, USA

## Abstract

This study characterized the time-location pattern of 107 residents living in air pollution hotspots, the Waterfront South and Copewood/Davis Streets communities in Camden, NJ. Most residents in the two communities are minority and impoverished individuals. Results showed that employment status played the fundamental role in determining time-location patterns of this study population, and the variations of time-location pattern by season and by day-type were partially attributed to employment status. Compared to the National Human Activity Pattern Survey, the Camden cohort spent significantly more time outdoors (3.8 hours versus 1.8 hours) and less time indoors (19.4 hours versus 20.9 hours) than the general US population, indicating a higher risk of exposure to ambient air pollution for the Camden cohort. The findings of the study are important for understanding exposure routes and sources for the socioeconomically disadvantaged subgroup and ultimately help develop effective strategies to reduce community exposure to ambient air pollution in “hotspots”.

## 1. Introduction

Time-location data are often required to adequately characterize human exposure and identify important locations where exposure occurred [1, 2]. They are also required inputs to estimate personal exposure in many models. For example, both the Hazardous Air Pollutant Exposure Model (HAPEM) [3] and the Air Pollutants Exposure Model (APEX) [4] utilize individuals' activity patterns in combination with concentrations of air pollutants in microenvironments of interest to estimate exposure levels. Time-location data have been collected in many human activity surveys and human exposure studies, and some have been built in the Consolidated Human Activity Database (CHAD) developed by USEPA [5]. Studies have shown that age, gender, season/temperature, day-type (work/nonwork or weekday/weekend), and employment status are the major factors that influence human time-activity-location patterns [6–8]. 

However, previous studies collected time-location data mostly by telephone interview, which would overlook the minority and the populations living in poverty who often do not have telephones. Thus, the information collected by those studies may not be representative for the socioeconomically disadvantaged group [9]. As documented in the U.S. Census data (2000), the poverty rate for minorities in the U.S. is as high as 21.9%, compared to 12.6% of the total U.S. population. In particular, 24.9% of African Americans and 21.8% of Hispanics live below the poverty threshold. Minorities and the impoverished tend to live in communities that are close to air pollution sources [10, 11]. Many studies have repeatedly documented that pollution sources are disproportionately located in low-income communities [11–18] so that the residents of those communities, many of them are minority and poor, bear a greater impact from mobile and industrial air pollution sources when compared to general population [19]. Thus, it is critical to characterize the exposure-related time-location patterns of this subpopulation in order to understand their potential exposure routes and sources.

To fill some of the knowledge gaps, this study investigated the time-location patterns of the residents in the Waterfront South (WFS) and Copewood/David streets (CDS) neighborhoods in Camden, New Jersey. The WFS and CDS neighborhoods are minority-dominated low-income communities. Both have been confirmed to be air pollution hotspots that are subject to elevated air toxic levels [20]. The time-location data used in this study were collected in a “hotspot” study, “Personal and Ambient Exposures to Air Toxics in Camden, New Jersey”, which characterized personal exposures to a suite of air pollutants, including volatile organic compounds (VOCs), aldehydes, polycyclic aromatic hydrocarbons (PAHs), and PM_2.5_, in WFS and CDS [21]. The aim of this study is to identify the factors that influence the time-location patterns of the Camden study cohort, compare their time-location patterns with the U.S. general population, and determine whether the time-location patterns of the Camden study cohort may lead to a higher risk of exposure to ambient air pollution. 

## 2. Methods

### 2.1. Study Cohort

The WFS neighborhood measures 1.5 × 0.5 miles, with 27 industrial facilities identified in the neighborhood. The CDS neighborhood is a residential area located about 1 mile east of the WFS, about the same size as WFS, and there are no nearby (<1 mile) industrial facilities in CDS. Based on the 2000 U.S. Census (U.S. Census Bureau, 2005), ~85% of the residents living in the two neighborhoods are nonwhite minorities. Both communities have significantly higher percentage of African Americans, 61% in WFS and 72% in CDS, respectively, while the national average is only 12.9%. In addition, both communities have a relatively younger population, with a median age of ~27 years old compared to the national median of ~35 years. Further, both communities have ~33% of the population below the poverty level, much higher than the national average of 12.6%. 

A total of 107 subjects aged from 8 to 80 years participated in the study, with 54 subjects from WFS and 53 subjects from CDS. Only nonsmokers living in the designated areas were eligible. We worked with local community leaders in both neighborhoods to help advertise the study. A local liaison was also hired to conduct door-to-door recruitment in the target areas. Other approaches, for example, advertising in local newspapers and distributing study information during local community events, were also employed. In those economic-disadvantaged neighborhoods, face-to-face recruitment was particularly important, because some households cannot afford phone service. For those without phones, the contact information of their friends or relatives was obtained in case of emergency contact, and their sampling was scheduled in-person during field trip. It is necessary to note that the recruitment was not through a strict stratified randomization procedure. The consent form, questionnaires, and study protocols were approved by the Institutional Review Board (IRB) of the University of Medicine and Dentistry of New Jersey (UMDNJ) prior to the start of the study. 

### 2.2. Data Collection

The air sampling as well as time-location data collection were carried out by the staff from Exposure Science Division at UMDNJ during the summer (late June to early September) and winter (late November to early April) seasons between July 2004 and July 2007. 

**Figure 1:**
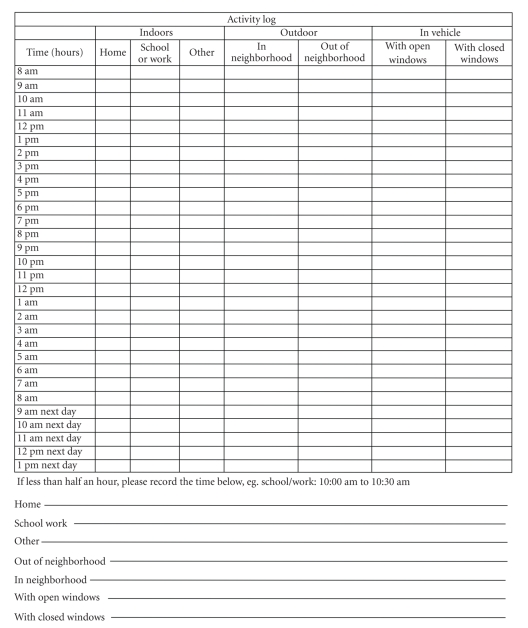
An example of time diary used in the Camden study.

Baseline Questionnaire and Time Diary/Activity Questionnaires were administrated to the subjects in the Camden study. The Baseline Questionnaire collected the basic demographic information on participant characteristics, socioeconomic status, housing condition, and simple respiratory health status of participants. Time-location data were collected by the Time Diary, which had seven microenvironments grouped into three categories, that is, home, work/school, other indoors in the indoor category, outdoors in the neighborhood and outdoors outside of the neighborhood in the outdoor category, and in vehicle with window open and in vehicle with window closed in the in-vehicle category (Figure 1). Note that we did not include subcategory of “work” under “outdoor” microenvironment because our study focused on the total exposure from outdoors, due to transit, work, and play. 

Participants were shown how to fill in the time diary for each microenvironment where they stayed more than 10 minutes during the 24 hours sampling period. The information recorded on the questionnaires was usually reviewed by the field staff at the end of the sampling day, and the information that was missing by the subject was usually recovered by the staff with the assistance from the subject. Each subject was visited 4 times, that is, two in the summer and two in the winter, with one weekday and one weekend day during each season. 

In total, 360 diaries were collected simultaneously with the measurements of personal exposure data of air toxics. Of these, 335 diaries, which had no more than 3 hours of missing information, were considered valid. The missing hours were made up by assuming that half of the missing hours were for the activities prior to the missing time period and half were from the activities after the missing time period. This may introduce some errors to the comparison. However, the percentage of diaries with missing hours was less than 15%, and thus, the errors was not expected to affect the comparison results significantly.

### 2.3. Data Analysis

#### 2.3.1. Variation of Time-Location Patterns

Variations by *location* (WFS/CDS), *season *(summer/winter), and *day-type *(weekday/weekend) were examined for the time-location patterns of the Camden study cohort. Since employment status was found to be an important factor to affect time-location patterns of the general population, subjects were divided into three types according to their socioeconomic status: school children (7~17 years old), employed adults (with either full-time or part-time employment; excluding self-employed subjects who work at home) and unemployed adults (including retirees). 

Given the focus on potential exposure to ambient air pollution in the hotspot, the time-location data collected by Time Dairies were regrouped. Total time that each subject spent indoors, outdoors, and in vehicle during the 24-hour monitoring period was calculated. The outdoor time spent in the neighborhood and the total time spent in the neighborhood was particularly emphasized. The time-location data were divided into subgroups by variables to be examined (i.e., *location, season,* and *day-type*), and the comparison by each variable was conducted between subgroups. Since the time-location data were not normally distributed, the Mann-Whitney-Wilcoxon test was used for comparison. 

#### 2.3.2. Comparison with the U.S. General Population

The National Human Activity Pattern Survey (NHAPS) [9] was conducted between 1992 and 1994 with support from the US EPA. The NHAPS collected 24-hour time-location-activity data by telephone interview from 9,386 respondents all through the U.S. (except Alaska and Hawaii), providing an overview of the time-location patterns of the U.S. general population. Klepeis et al. [9] also reported that the U.S. population spent fairly consistent time indoors over the past few decades. Therefore, it was used for comparison with the time-location patterns of the Camden study population.

As time-location data collected from an individual on multiple sampling days in the Camden study might be correlated, the intraclass correlation coefficient (ICC) was examined first to ensure that within-subject correlation would not significantly confound the comparison. A hierarchical mixed effects model was fit to apportion *season* and *day-type* variance nested within each participant. ICC is defined as the ratio of between-subject variance to total variance [22], and is computed for time spent in each microenvironment. An ICC of 80% indicates strong correlation among the measurements (i.e., multiple diaries in our study) collected from an individual. In contrast, a lower ratio corresponds to less correlation among the multiple diaries from the same individual, suggesting that the diaries collected from an individual are independent from each other. 

The microenvironment categories used in the NHAPS and the Camden study were not the same, thus the microenvironments of both studies were grouped into broader categories, that is, total time spent indoors, total time spent outdoors, and total time spent in vehicle(s), so that the data collected from both studies can be compared. The time-location data of the Camden study were weighted against the 2000 U.S. Census data for the WFS and CDS neighborhoods using the same weighting method employed in NHAPS [23], to account for the unrepresentative ratios of genders and age groups as well as disproportionate sampling by season and by day-type. 

For comparison, the time-location data of the Camden study were stratified into a series of subgroups by *gender*, *age group*, *season* and* day-type*, and a weighting factor was assigned to each subgroup. Data collected from the WFS and CDS neighborhoods were pooled for comparison with the NHAPS data. Three age groups were defined in this study: school age (5–17), working age (18–64), and senior (65 and over). The weighted average time spent in each microenvironment was calculated by *gender*, by *age group*, by *season,* and by *day-type*, and then compared with the weighted population mean calculated by Klepeis et al. [23]. Time-location data of both studies are highly skewed. However, given the large sample size in each comparison group (*N* > 100), the Central Limit Theorem was applied and the *T*-test was employed for comparison. 

Bonferroni correction was applied in the variation analysis of time-location patterns of the Camden cohort and the comparison with the U.S. general population, to control the overall significance level of multiple comparisons. SAS for windows version 9.1 (SAS Institute Inc., Cary, NC, U.S.) was used for all analyses in this study.

## 3. Results

The demographics of the Camden study cohort are similar to the entire neighborhood population (Table 1). Females were oversampled 10%–20%, and this would be adjusted in the comparison with NHAPS. The time-location data collected in the Camden study are summarized in Table 2 by season and by day-type for each type of subjects in the WFS and CDS communities. Briefly, people living in these neighborhoods spent the majority of their time in the neighborhood (18.2~23.6 h), more time indoors (16.8~22.3 h) than outdoors (1.4~6.3 h). Given their routine work, the employed adults spent significantly more time at work and in the vehicle, and much less time in the neighborhood on weekdays than the school children and unemployed adults. Time-location patterns on weekends tended to be similar among these three types of subjects. More than half of the school children and unemployed adults reported spending no time in vehicle. Below we presented the variation of the time-location patterns of the Camden cohort and a comparison with the general U.S. population.

### 3.1. Variation of Time-Location Patterns

#### 3.1.1. Location

Under most circumstances, there was no statistically significant difference in time-location patterns between the participants living in the WFS and CDS communities, which is consistent with the geographic locations and demographic information of the two communities. As introduced earlier, the WFS and CDS neighborhoods are located only one mile away from each other, and these two communities have similar demographic constitution and economic status. The time-location patterns of the residents from these two neighborhoods, therefore, were expected to be similar. 

However, we observed marginally significant difference (*P* = .014) in time spent in vehicle between WFS and CDS, where the employed adults in CDS spent more time in vehicle than those in WFS on the summer weekdays. We also observed the differences in time-location patterns among the three types of subjects only from the CDS not WFS, for example, the employed adults spent less time outdoors than the school children and the unemployed adults on summer weekdays, and the unemployed adults spent more time at home on winter weekday. This may be due to the difference in employment status of the subjects between the two neighborhoods, that is, more employed adult subjects in CDS had full-time jobs rather than part-time jobs, while it was opposite in WFS.

#### 3.1.2. Season

Significant seasonal variations of time-location patterns were observed for the school children and unemployed adults in the CDS neighborhood on weekdays. These two subgroups spent more time outdoors (*P* = .004 for school children and *P* = .015 for the unemployed adults), particularly in the neighborhood, in summer than in winter. School children also spent more time at school (*P* < .006) on weekday in winter than in summer, because our summer sampling season overlapped the school summer breaks. In contrast, no seasonal variation of time-location patterns was observed for the employed adults. 

An unexpected finding of this study is that the time-location patterns of the WFS residents did not vary significantly by season. Take the *total time spent outdoors* as an example, subjects in WFS consistently spent a large amount of time outdoors on weekdays in both summer and winter: 3.6 hour/day (median, same hereafter) in summer and 2.9 hour/day in winter for children (*P* = .75), and 4.6 hour/day in summer and 4.1 hour/day in winter for the employed adults (*P* = .61). These observations suggested a unique time-location pattern of the WFS cohort, which would increase the chance of exposure to elevated ambient air pollution in the hotspot neighborhood.

#### 3.1.3. Day-Type (Weekday versus Weekend)

As expected, the school children and employed adults spent significantly more time at school/work during weekdays than on weekend days in the winter. School children in the CDS neighborhood spent more time indoors and less time outdoor (*P* < .004) during the weekday than on the weekend in winter. Time-location patterns of the unemployed adults did not vary by *day-type*. 

### 3.2. Comparison with the U.S. General Population

As presented above, the time-location patterns of the participants living in the WFS and CDS communities were similar under most of the circumstances, thus data collected from these communities were combined when compared with the NHAPS data.

Prior to comparison, the ICCs were evaluated to examine whether the correlation among the diaries collected from each individual confounded the comparison. The ICCs ranged from 0.05 to 0.36 were less than 0.30 for most of the microenvironments, for example, 0.17 of the total variance of time spent indoors and 0.15 of the total variance of time spent outdoors were due to individual variability. The ICC for the total time spent in vehicle was slightly higher (0.29), which might be attributed to subjects' employment status and possession of vehicle. Given the low intrasubject correlation, multiple time-location data collected from the same individual could be considered independent in the following analyses.

We cross-checked the gender and age constitutions of the Camden cohort with the census data of these neighborhoods (U.S. Census 2000) and the U.S. general population (U.S. Census 1990) (Table 3). It is found that we moderately oversampled females aged 5–17 years and 18–64 years, and undersampled the senior group (65+) in the WFS and CDS neighborhoods. No statistical comparison was conducted for the senior group due to the small sample size. Compared to the U.S. general population, population in the WFS and CDS neighborhoods were younger and had more females. 

**Figure 2:**
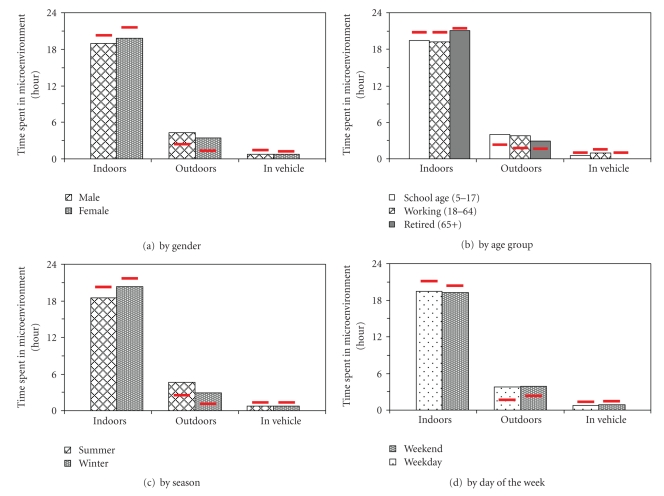
Comparison of the weighted mean percentages of time spent in each microenvironment between the Camden study cohort (*N* = 335) and the U.S. general population (*N* = 9368) [9]. (Red bars are the weighted NHAPS means, which represent the general U.S. population levels.)

Significant differences in time-location patterns were observed between the Camden study cohort and the NHAPS data, which represents the time-location patterns of the U.S. general population (Figure 2). The Camden cohort spent significantly more time outdoors and less time indoors and in vehicle(s) than the U.S. general population (*P* < .001), for both genders and two age groups (children and adults at working age), in both seasons and on both weekdays and weekend days. In particular, the Camden study cohort spent a large amount of time outdoors (weighted average: 15.8%, 3.8 h), which is more than two times of the national average level (7.6%, 1.8 h). Results of the senior group also showed the same tendency, though in a small sample size. 

The variations of time-location patterns by gender, age, season, and day-type for the Camden cohort were basically consistent with those of the NHAPS, that is, males spent more time outdoors and less time indoors than females among the Camden cohort, and people spent more time outdoors and less time indoors in summer than in winter. However, the variation by day-type observed in the NHAPS, that is, people spent more time indoors on weekdays and less time indoors on the weekend, was not observed in the Camden data. There was no significant difference in time spent in vehicles by season or by day-type for either the Camden cohort or the U.S. general population.

To further explore the unique time-location patterns of the Camden cohort, the larger amount of time spent outdoors relative to indoors, the percentages of time spent at home and indoor work environments were compared with NHAPS. Among the U.S. general population, the working age adults spend an average of 3.3 hours at work or school (including both indoor and outdoor work). In contrast, the working age adults in the Camden study cohort spent much less time at work (indoor environment only), only 54 minutes per day. They spent 1.7 hours more at home but 2.3 hours less indoors at work/school, thus, the net difference in time spent indoors would be 0.6 hours less than the U.S. general population. These observations were probably related to the low-employment rate and poor job stability among our study population. The fact that many people were part-time workers or engaged in outdoor tasks may also contribute to the exceptional higher outdoor time observed in this population.

## 4. Discussion

This study characterized the time-location patterns of the Camden study cohort, a minority-dominant and impoverished population living in air pollution hotspots. This population has a different time-location patterns from the U.S. general population, with particularly longer time spent outdoors in the neighborhoods. The elevated neighborhood concentrations of air toxics and the large amount of time spent outdoors likely place this disadvantaged subgroup at higher risks of exposure to ambient air pollution and thus adverse health effects than the U.S. general population. Given the concerns and the knowledge gaps, there is a pressing need to conduct a comprehensive study to characterize the time-location patterns of this subpopulation in order to understand their exposure routes and potential sources of exposure to ambient air pollution. 

Previous studies have recognized that employment status is an influential factor on time-location patterns among the general population [6–8, 24]. It was considered one of the secondary factors behind season and day-type in explaining the variation of time-location patterns [7]. However, our study shows that employment status plays the fundamental role in determining the time-location patterns of the Camden cohort. For example, the unemployed adults and school children spent more time indoors in winter or more time outdoors in summer, which is the basic trend observed in other human activity studies, for example, NHAPS [9], but this was not observed for the employed adults. The school children and employed adults consistently spent more time at work/school on weekdays, and spent more time at home and/or outdoors on weekend days, while the time-location patterns of the unemployed adults did not show any day-type difference. In Camden, employment status determined the flexibility of one's time budget, and thus fundamentally influenced the variation of time-location patterns for our study cohort.

The employment rates in the WFS and CDS communities were very low, especially with respect to the full-time employment (Table 1), which was consistent with the well-established correlation between minority group and employment disadvantage [25]. Comparison with the NHAPS data also demonstrated the impact of the employment rate on time-location patterns. The Camden study cohort spent exceptionally less time at indoor work environment but significantly more time outdoors compared to the U.S. general population, though they might work outdoors. Further, 50% of the employed adults in the Camden cohort had a part-time job, much higher than the percentage (~16%) of the NHAPS cohort. The percentage of subjects with part-time jobs could be an important factor to explain the differences in time-location patterns between these two studies. Moreover, according to the census data, 74% and 58% households in the WFS and CDS neighborhoods had vehicle(s), much lower than the national average level of 90%. The low employment rate and low vehicle possession rate for the Camden cohort probably explained their low percentage of time spent in vehicles. 

Besides the NHAPS, most of the previous human activity studies reported shorter time spent outdoors than those observed for the Camden study cohort. For example, Quackenboss et al. [26] reported that time spent outdoors accounted for 3.3 hours per day in summer and 0.8 hours per day in winter in Wisconsin, and Echols et al. [24] reported that time spent outdoors was 2.2 hours in summer and 1 hour in winter in Maryland. In contrast, the Camden study cohort spent 4.8 hours in summer and 2.8 hours in winter outdoors. These differences may be partially attributed to different geographic location of each study group, but more importantly different socioeconomic status of the study cohort. 

The Camden subjects spent considerably more time outdoors compared to the NHAPS and other human activity studies. The disadvantaged socioeconomic status was the predominant reason. However, the differences in study design of these two studies may also contribute partially to the differences of the time-location patterns. First, the Camden study cohort did not cover young children less than 5 years old and undersampled the elders above 64 years old, which was 10%–15% of the population in the WFS and CDS neighborhoods. These two subpopulations spent a considerable time indoors, thus we may underestimate the population-average time spent indoors. Second, the Camden study collected time-location data using self-administrated time diaries during field sampling period, while NHAPS relied on recall during phone interviews. McCurdy and Graham [7] pointed out that a diary usually collects more accurate information than recall, and data collected by telephone recall usually miss the time spent in transit between different locations, thus may underestimate the time spent outdoors and in vehicle(s) in NHAPS. Further, one should note that the “in-vehicle” defined in the Camden study is different from the “in-transit” in some human activity studies [24, 27]. Outdoor transit activities, for example, walking and biking, were considered “in-transit” but are not “in-vehicle”. Thus, we expected slightly longer time spent outdoors but shorter time spent in vehicle(s) in the Camden study when compared with those studies. Given above, we expect underestimation of time spent indoors for our study cohort to certain extent but those estimation errors are not large enough to change the differences between the Camden data and the NHAPS. 

In conclusion, given the low-employment rate in the WFS and CDS neighborhoods, a large number of residents, including children and unemployed adults, spent 93~100% (median) of their time in the neighborhood in both summer and winter seasons, independent of day-type, compared to 75~92% of the time spent by the employed adults in the neighborhood. Considering the WFS and CDS neighborhoods as hotspots of air pollution, the school children and unemployed adults in these two neighborhoods bear a greater potential for exposure to local ambient air pollution and thus adverse health effects. Furthermore, the participants at school/working age of the Camden study spent significantly more time outdoors and less time indoors and in vehicle(s) than the U.S. general population in both winter and summer, on weekdays and weekend days. Given the particular time-location patterns of the Camden cohort and the elevated air pollution of their neighborhoods, Camden residents, and especially the disadvantaged individuals, are more likely to experience higher exposure to air pollution than the U.S. general population. 

There are several limitations of the study. First, the sample size is relatively small, thus, verification of our findings in a larger study cohort is needed. Second, limited to the participation of our study, oversampling of females and undersampling of seniors occurred, which may affect the representativeness of the time-location patterns for the entire community population. However, to avoid potential bias in comparison, we grouped subjects by employment status when examining the variation of time-location patterns, and used the weighted means when comparing with the U.S. general population. Despite of the limitations, this study provides important time-location data for the minority and impoverished population living in air pollution hotspot areas. These data will help to identify potential exposure routes and sources for this subpopulation and aid in developing effective strategies to reduce their exposures to air pollution. 

## Figures and Tables

**Table 1:** Demographics of the Camden study cohort and the comparison with the U.S. Census.

	U.S. Census^(a)^		The Camden Study^(b)^	
	WFS	CDS	WFS	CDS
*N*	1,700	6,424	54	53
Gender				
Male	49.2%	45.6%	42.6%	28.3%
Female	50.8%	54.4%	57.4%	71.7%
Age^(c)^				
Under 5 years	8.8%	9.4%	—	—
5 to 17 years	27.4%	26.7%	29.6%	34.0%
18 to 64 years	55.5%	57.0%	64.9%	64.2%
65 years and over	9.2%	7.0%	5.6%	—
Median age (years)	27	26.7	29	24.5
Race and Ethnicity^(d)^				
White	17.3%	14.0%	20.4%	1.9%
Black or African American	61.2%	71.5%	51.9%	71.7%
American Indian and Alaska Native	1.3%	0.9%	—	—
Asian	6.5%	0.7%	—	—
Native Hawaiian and Other Pacific Islander	0.1%	<0.1%	5.6%	—
Refused/Do not know			22.2%	26.4%
Hispanic or Latino (of any race)	27.2%	25.6%	27.8%	20.8%
Education^(e)^				
High school graduate or higher	49.4%	57.4%	48.1%	39.6%
Bachelor's degree or higher	4.2%	5.5%	11.1%	7.5%
Refused/Do not know			38.9%	43.4%
Employment status^(f)^	26.1%	31.5%		
Adult working full time	N/A	N/A	13%	20.8%
Adult working part time	N/A	N/A	22.2%	1.9%
Self employed working at home	N/A	N/A	5.6%	9.4%
Refused/Do not know			9.3%	22.6%
Median household income ($)^(g)^	22,417	25,443	N/A	N/A
Income below poverty level^(g)^	32.7%	33.3%	N/A	N/A

^(a)^Data are from U.S. Census 2000.

^(b)^A  dash indicates no subject in that group.

^(c)^Age  of the Camden study cohort was determined as of 2005. One subject in WFS had age missing.

^(d)^Some  Camden subjects selected multiple answers in responding to the question about cultural background. Data in the table were based on the primary choice.

^(e)^Percentages  in the Census were based on the population aged 25 and over, while percentages in the Camden study cohort were based on population aged 18 and over.

^(f)^Employment  rate in Census was based on the population above 16 years old; while the one of the Camden study was based on the population above 18 year old.

^(g)^Census  data were collected in 1999.

**Table 2:** Time spent in microenvironments (hour) by subject type, season and day-type in the WFS and CDS neighborhoods.

Category	Summer								Winter							
	Weekday				Weekend				Weekday				Weekend			
	Mean	SD	Med	95th %	Mean	SD	Med	95th %	Mean	SD	Med	95th %	Mean	SD	Med	95th %
School Children	(*N* = 28)				(*N* = 25)				(*N* = 30)				(*N* = 29)			
Home	17.8	3.8	17.6	24.0	19.1	4.1	20.4	24.0	16.3	3.7	15.6	24.0	16.8	4.7	17.0	22.6
Work/School	0.0	0.0	0.0	0.0	0.0	0.0	0.0	0.0	3.8	3.8	3.8	8.0	0.5	1.6	0.0	5.1
Outdoors in the neighborhood	4.2	3.5	3.6	10.6	3.4	4.2	1.1	11.5	1.8	2.7	1.0	10.0	3.4	2.8	2.9	8.6
Total in the neighborhood	22.3	2.8	24.0	24.0	22.7	2.7	24.0	24.0	22.1	2.9	24.0	24.0	21.5	4.0	24.0	24.0
Total indoors	18.3	3.9	18.0	24.0	19.3	4.1	20.4	24.0	20.8	3.0	21.8	24.0	18.9	3.7	20.0	23.0
Total outdoors	5.3	3.7	5.7	11.0	4.1	4.1	2.9	11.5	2.7	2.9	1.9	10.0	4.1	3.2	3.4	10.6
Total in-vehicle(s)	0.4	0.9	0.0	2.5	0.6	1.9	0.0	3.3	0.5	1.1	0.0	3.3	1.0	2.3	0.0	6.0

Employed Adults	(*N* = 27)				(*N* = 27)				(*N* = 23)				(*N* = 21)			
Home	14.9	5.6	14.0	24.0	17.3	6.0	19.0	24.0	15.7	5.6	17.2	22.0	17.8	3.4	17.8	22.6
Work/School^(a)^	1.9	3.6	0.0	9.0	0.3	1.3	0.0	2.0	2.7	4.0	0.9	9.5	0.4	1.7	0.0	0.2
Outdoors in the neighborhood	3.0	4.3	1.9	10.6	2.4	3.1	1.6	7.4	2.0	2.3	1.0	6.0	1.6	2.6	0.0	5.8
Total in the neighborhood	19.3	4.5	20.0	24.0	20.8	4.7	23.0	24.0	19.1	6.5	21.9	24.0	20.1	2.9	20.0	24.0
Total indoors	18.1	4.7	18.5	24.0	19.0	4.2	20.0	24.0	19.6	2.7	19.7	23.5	18.5	3.6	18.7	23.0
Total outdoors	4.1	4.6	4.0	11.5	3.8	3.9	2.8	12.0	3.1	2.8	2.6	6.2	3.9	3.8	2.0	12.0
Total in-vehicle(s)	1.8	2.0	1.3	5.5	1.2	1.5	0.8	4.3	1.3	1.4	1.0	3.8	1.6	1.9	0.9	5.0

Unemployed Adults	(*N* = 34)				(*N* = 32)				(*N* = 30)				(*N* = 29)			
Home	18.0	4.1	18.3	24.0	17.8	4.8	18.4	24.0	19.7	4.3	20.3	24.0	19.0	4.1	20.0	24.0
Work/School^(b)^	0.0	0.0	0.0	0.0	0.0	0.0	0.0	0.0	1.1	2.5	0.0	6.7	0.4	1.5	0.0	3.0
Outdoors in the neighborhood	3.5	2.9	3.4	9.5	2.9	3.2	1.7	9.0	1.8	2.5	0.4	6.5	2.0	2.6	1.0	7.7
Total in the neighborhood	22.3	2.5	24.0	24.0	21.6	3.2	23.0	24.0	22.4	3.7	24.0	24.0	21.9	3.1	24.0	24.0
Total indoors	18.8	3.7	18.9	24.0	18.8	4.1	19.1	24.0	21.2	3.2	22.8	24.0	20.2	3.3	21.0	24.0
Total outdoors	4.7	3.7	4.6	10.7	4.6	3.9	3.7	12.5	2.4	3.0	1.0	7.7	3.1	2.9	2.8	10.0
Total in-vehicle(s)	0.5	0.9	0.0	2.9	0.7	1.4	0.0	3.2	0.4	0.7	0.0	1.9	0.7	1.7	0.0	5.5

Note: SD: standard deviation; Med: median, 95th%: the 95th percentile.

^(a)^The median of time spent at work by employed adults was zero on weekday in summer, because some subjects' jobs were temporary or seasonal, for example, school teacher.

^(b)^Unemployed adults include college students. Unemployed adults may also work temporarily.

**Table 3:** Cross-check of the age and gender constitution (%) of Camden cohort with the census data (U.S. Census Bureau, 1990, 2000).

Age Group			Population in the WFS and CDS Census Tract (*P_h_*)		U.S. General Population	
	Camden Cohort (*p_h_*)		(U.S. Census Bureau 2000)		(U.S. Census Bureau 1990)	
	(*N* = 335)		(*N* = 8,087)		(*N* = 248,709,873)	
	Males	Females	Males	Females	Males	Females
0–4	—	—	5.0	3.9	3.8	3.6
5–17	14.0	17.6	14.1	13.1	9.3	8.9
18–64	20.6	44.5	23.4	33.2	30.6	31.3
65+	1.2	2.1	3.0	4.3	5.1	7.5

Total	35.8	64.2	45.6	54.4	48.7	51.3

Note: Weight *w*_*h*_ = *P*_*h*_/*p*_*h*_.
